# Intergenerational Predictors of Birth Weight in the Philippines: Correlations with Mother’s and Father’s Birth Weight and Test of Maternal Constraint

**DOI:** 10.1371/journal.pone.0040905

**Published:** 2012-07-27

**Authors:** Christopher W. Kuzawa, Dan T. A. Eisenberg

**Affiliations:** 1 Department of Anthropology, Northwestern University, Evanston, Illinois, United States of America; 2 Cells 2 Society: The Center for Social Disparities and Health at the Institute for Policy Research, Northwestern University, Evanston, Illinois, United States of America; 3 Department of Anthropology, University of Washington, Seattle Washington, United States of America; Indiana University, United States of America

## Abstract

**Background:**

Birth weight (BW) predicts many health outcomes, but the relative contributions of genes and environmental factors to BW remain uncertain. Some studies report stronger mother-offspring than father-offspring BW correlations, with attenuated father-offspring BW correlations when the mother is stunted. These findings have been interpreted as evidence that maternal genetic or environmental factors play an important role in determining birth size, with small maternal size constraining paternal genetic contributions to offspring BW. Here we evaluate mother-offspring and father-offspring birth weight (BW) associations and evaluate whether maternal stunting constrains genetic contributions to offspring birth size.

**Methods/Principal Findings:**

Data include BW of offspring (n = 1,101) born to female members (n = 382) and spouses of male members (n = 275) of a birth cohort (born 1983–84) in Metropolitan Cebu, Philippines. Regression was used to relate parental and offspring BW adjusting for confounders. Resampling testing was used to evaluate whether false paternity could explain any evidence for excess matrilineal inheritance. In a pooled model adjusting for maternal height and confounders, parental BW was a borderline-significantly stronger predictor of offspring BW in mothers compared to fathers (sex of parent interaction p = 0.068). In separate multivariate models, each kg in mother’s and father’s BW predicted a 271±53 g (p<0.00001) and 132±55 g (p = 0.017) increase in offspring BW, respectively. Resampling statistics suggested that false paternity rates of >25% and likely 50% would be needed to explain these differences. There was no interaction between maternal stature and maternal BW (interaction p = 0.520) or paternal BW (p = 0.545).

**Conclusions/Significance:**

Each kg change in mother’s BW predicted twice the change in offspring BW as predicted by a change in father’s BW, consistent with an intergenerational maternal effect on offspring BW. Evidence for excess matrilineal BW heritability at all levels of maternal stature points to indirect genetic, mitochondrial, or epigenetic maternal contributions to offspring fetal growth.

## Introduction

Individuals with lower birth weights (BW) have heightened risk of mortality in infancy and early childhood [1]. In the past few decades, a large literature has extended the health impacts of poor birth outcomes by showing that adults born small tend to have higher blood pressure, risk for diabetes, and cardiovascular disease morbidity and mortality [2], [3]. In addition, there is growing interest in the importance of fetal exposures, including nutritional stress and growth restriction, as programming cues for developmental plasticity [4].

Because fetal growth serves as a marker of prenatal conditions that influence an array of later biological functions and health outcomes, there is a need to understand the causes of variation in fetal nutrition, growth and birth outcomes. Estimates of heritability for BW typically range between 0.2 and 0.4 [5], [6], [7], with a recent study of all births in Norway between 1967–2004 finding that genetic factors accounted for 31% of BW variation [8]. In most populations, the majority of BW variation is believed to trace to maternal factors that influence the gestational metabolic environment, or by placing physical constraints on viable fetal size [9]. In a classic study, Walton and Hammond [10] used artificial insemination to reciprocally cross Shetland ponies and full-sized Shire horses. They found that newborn size was largely set by the breed and size of the surrogate mother rather than of the genetic mother, leading to the concept of “maternal constraint” e.g. that maternal phenotype, rather than fetal genotype, is most important in setting limits on growth attainment *in utero*. In this model, paternal factors were more important as influences on postnatal growth and final size.

In humans, evidence for a disproportionate maternal contribution to birth outcomes comes from several sources. Studies have compared the strength of father-offspring and mother-offspring BW correlations (e.g. [11], [12], [13], [14], [15]) and most report a stronger maternal than paternal effect on offspring BW [8], [12], [14], [15]. The causes of this matrilineal excess in BW heritability remains poorly understood mechanistically, but could reflect some combination of intergenerational influences of gestational conditions, as illustrated by transgenerational epigenetic effects on birth size in animal model experiments [16], sex-linked genetic effects, indirect maternal genetic effects [17], mitochondrial effects [18], shared family effects, or false paternity [19].

In addition to evidence for stronger intergenerational BW correlations through the matriline, a related hypothesis posits that genetic contributions to BW are constrained in women who experienced early life histories of nutritional stress [20]. Ounsted et al (1986) found evidence that genetic contributions to offspring BW were stronger among matrilines characterized by normal BW, with attenuated effects among women who were themselves born as small babies. Evidence for such effects would support the idea that a stressful maternal nutritional history, starting *in utero*, could override genetic influences on the prenatal nutritional environment experienced by the next generation and be reflected in fetal growth rate and birth size. This idea has gained limited support in some [21] but not all studies, and has been critically reviewed [22]. As perhaps the only prior test of this hypothesis in a lower income setting, a recent study of an Indian population in which nutritional problems were relatively common failed to confirm the expectations of this model [13].

Here we contribute to the literature on genetic and environmental contributions to BW by reporting the strength of mother-offspring and father-offspring BW associations in a well-characterized birth cohort in Cebu City, the Philippines. As part of this analysis, we run a resampling analysis in an effort to determine the false-paternity rate that would be needed to account for any differences in matrilineal and patrilineal intergenerational correlations in this sample. Finally, we test for maternal constraint on fetal growth rate by evaluating whether the strength of parental-offspring BW correlations is stronger among heavier babies that were less likely growth-restricted *in utero*, and whether the intergenerational BW correlations are reduced among women who were shorter as adults.

## Materials and Methods

Data come from the Cebu Longitudinal Health and Nutrition Survey (CLHNS), a population-based study that originally enrolled 3327 pregnant mothers and has since followed their offspring into adulthood, many of whom are now parents with offspring of their own [23]. In the present analyses, we relate prospectively-measured BW and gestational age obtained at birth in fathers and mothers (male and female birth cohort members born in 1983–84) to recalled BW and gestational timing information obtained for their offspring (born between 1999 and 2009). The variables used in the present analysis and their collection methods are described in Table 1. This research was conducted with written informed consent of all participants and with human subjects clearance from the Institutional Review Boards of Northwestern University and the Office of Population Studies Foundation (University of San Carlos, Cebu).

**Table 1:** Variable collection methods.

Variable	Prospective/Recalled
Participants (born 1983–4)	
Birth weight	Prospective
Gestational age	Prospective
Current height	Prospective
Adult household income	Prospective
Spouses of male participants	
Height	Prospective
Weight	Prospective
Offspring (of 1983–4 born parent)	
Birth weight	Recalled (maternally)
Gestational age	Recalled (maternally)
Twins (Yes/No)	Recalled (maternally)
Birth date	Recalled (maternally)
Weighed (Yes/No)	Recalled (maternally)
Live birth (Yes/No)	Recalled (maternally)
Weight	Recalled (maternally)
Gestational age	Recalled (maternally)
Sex	Recalled (maternally)
Mother worked in pregnancy	Recalled (maternally)
Mother received prenatal care	Recalled (maternally)
First born	Recalled (maternally)

a Male and female – mothers and fathers.

### Offspring Birth Outcomes

Offspring BW information was obtained through recall among female birth cohort members and among the current spouses of male cohort members. Each mother was asked to recall, among other factors, the status of each pregnancy (twin, singleton, liveborn, stillbirth), birth date, whether the baby’s weight was weighed and if so the BW, gestational age at parturition, and sex of each of her offspring. Because BW is reduced in twin pregnancies, and twin pregnancies were rare, analyses were limited to singleton liveborn pregnancies. In addition to birth outcome characteristics, we obtained information on characteristics of each pregnancy, including whether the woman worked while pregnant and whether she obtained prenatal care. Current height and weight were obtained using standard anthropometric techniques [24].

Of the 1235 singleton liveborns for which all maternal and birth control variables were available, the baby was not weighed at 134 of these births (10.9%) and these pregnancies were therefore excluded from analysis. We compared women whose babies were weighed at birth to those whose baby was not. Women who did not have their baby weighed at birth came from lower income households (336±476 pesos vs. 504±540 pesos, linear regression, p<0.0001), they were slightly less likely to receive prenatal care (96% vs. 99% in those weighed, logistic regression, p<0.01), and they were also less likely to report having worked during pregnancy (24% vs. 41% in those with babies weighed at birth, logistic regression, p<0.001). However, women whose baby was not weighed had a similar adult stature to women whose baby was weighed at birth (150.3±5.7 cm vs. 151.3±5.4 cm, linear regression, p<0.25).

### Statistical Analyses

All analyses were performed with Stata version 10 (College Station, TX), except the paternal uncertainty analyses which were conducted in R (see File S1 for details.) We began by describing the characteristics of the mothers (female ’83–84 born cohort members and female spouses of male cohort members) and of the individual births. We then built a series of regression models predicting offspring BW which were run separately for cohort females and female spouses of male cohort members. Because some mothers had had multiple offspring, we used the regress command with the cluster option (clustering on mother) in Stata to adjust for the non-independence of data points in multiparous women. We began with a base model predicting BW that adjusted for birth-specific characteristics, including mother’s age during that pregnancy, primiparity status, gestational age at birth (two dichotomous variables delineating whether the mother reported that the baby was “born early” or “born late”, with “on time” being the comparison group), sex of baby, and whether the mother worked or received prenatal care during that pregnancy. To the base model, we then sequentially added the parent’s BW (the mother’s own BW for offspring of female cohort members, the father’s BW for offspring of their spouses), then the parent’s gestational age at birth, and finally, the mother’s adult height. We also tested an interaction between the parent’s BW and the mother’s adult height to test whether intergenerational BW associations are attenuated among shorter women or among women showing evidence for nutritional stunting.

**Figure 1:**
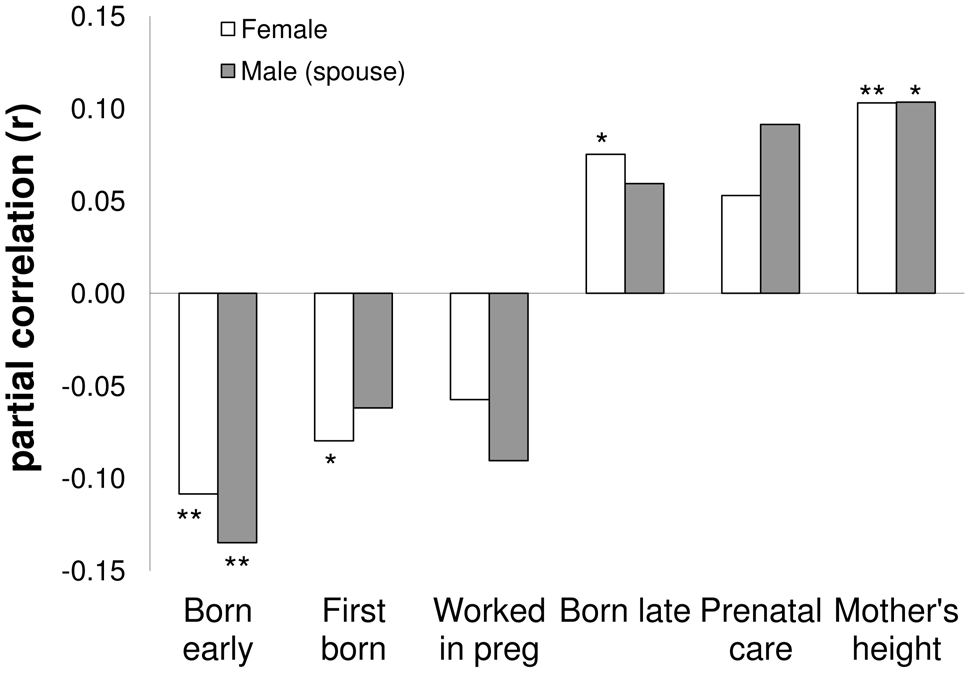
Pearson’s correlation coefficients relating maternal and pregnancy characteristics to BW among female participants and spouses of male participants. * p<0.05 ** p<0.001.

Any differences in the strength of association between paternal and maternal BW could be secondary to differences in the reliability with which female participants and spouses of male participants recalled the BW of their offspring. The similar strength of correlations between a range of maternal predictors and recalled BW in both female participants and spouses of male participants (**Fig. 1**) suggest that measurement reliability is similar for female participants and spouses of male participants.

## Results

Descriptive characteristics of the two groups of mothers – the female cohort members and the spouses of male cohort members – are reported in Table 2. Spouses of cohort males tended to be slightly taller than female cohort members, but had similar household income. Birth weights of female cohort member mothers and male cohort member fathers were similar. Pregnancy characteristics of the two groups of mothers were generally similar (Table 3). The spouses of male cohort members were on average several months older at the birth of their offspring compared to female cohort members, and their baby was about 8% more likely to be a first born. Among these women, 10% fewer reported working during pregnancy, compared to the female cohort members.

**Table 2:** Characteristics of parents (birth and adulthood).

	Female participants (n = 382)	Spouses of male participants (n = 275)	p-value
Mother’s height (cm)	150.8 (5.3)	152.2 (5.5)	0.00001
Adult household income^a^	492 (517)	519 (573)	0.996
Mother’s birth weight (kg)	3.0 (0.4)	–	
Father’s birth weight (kg)	–	3.0 (0.5)	

Values are mean (SD).

a n = 629.

**Table 3:** Characteristics of individual pregnancies.

	Offspring of female participants (n = 675)	Offspring of male participants (n = 426)	p-value
Mother’s age at birth (years)	21.8 (2.3)	22.2 (3.2)	0.032
Birth weight (kg)	2999 (556)	3014 (509)	0.683
Gestational age (months)	9.0 (0.2)	9.0 (0.3)	0.293
Male (%)	55.0%	50.7%	0.169
First born (%)	56.6%	64.6%	0.001
Mother worked in pregnancy (%)	45.0%	34.7%	0.004
Mother received prenatal care (%)	99.1%	99.5%	0.552

Values are mean (SD) unless noted otherwise.

We next ran regression models relating parental BW with offspring BW with both samples pooled (not shown). In a model predicting birth outcomes in female cohort members and spouses combined, there was a borderline sex of parent by parental BW interaction (p = 0.068), showing that maternal and paternal BW relate to offspring BW with (borderline) different slopes. All subsequent models were stratified on gender of the parent for whom BW was measured.

**Figure 2:**
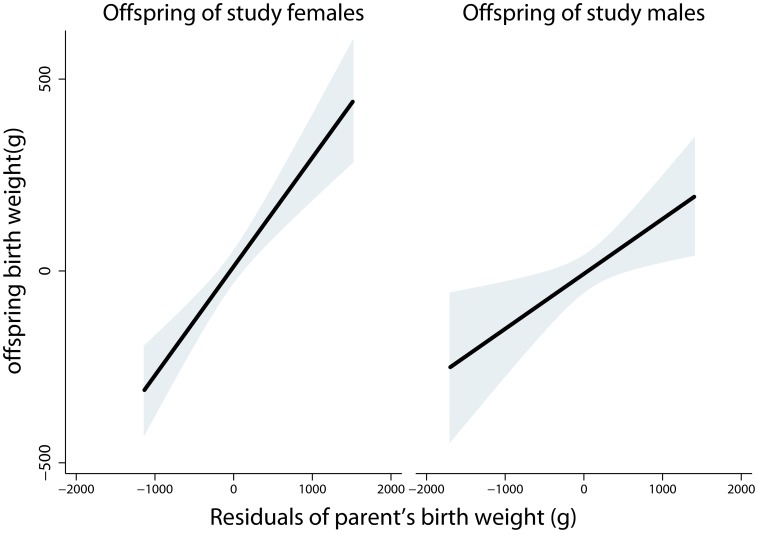
Best fitting regression lines (shaded area = 95% CI) relating offspring BW to maternal BW (n = 675) and father’s BW (n = 426). Parental BW plotted as residuals adjusted for parent’s gestational age at birth. Offspring BW plotted as residuals adjusted for mother’s age, offspring’s gestational age at birth, first born status, offspring sex, prenatal care, and mother’s work status during pregnancy (sex of parent by parental BW interaction p = 0.074).

Tables 4 and 5 report a series of 4 models which were run separately for female cohort members and female spouses of male cohort members. Mother’s age, primiparity status, timing of delivery, offspring sex, prenatal care and work status during pregnancy together explained roughly 3% of the variance in offspring BW among both groups of mothers (Model 1). Adding mother’s BW more than doubled the variance explained in BW of offspring born to female cohort members, while adding father’s BW to models predicting offspring of their spouses made more modest contributions to explained variance (Model 2). The regression coefficient linking mother’s BW to offspring BW, reflecting the unit change in offspring BW (grams) predicted by a unit change in parental BW (kilograms), was twice as steep as the relationship between father’s BW and offspring BW. These relationships did not significantly vary by the sex of the offspring for either female or male parental generation cohort members (BW by Sex interaction of p = 0.143 or p = 0.707 respectively; models not shown). Adjusting for parent’s gestational age at birth (Tables 4 and 5: Model 3) strengthened the relationship with BW for both mothers and fathers, showing that fetal growth rate is an important predictor of offspring BW in this sample. Finally, adjusting for maternal stature (Model 4) attenuated these relationships slightly, although both remained significant. Figure 2 plots best-fitting regression lines relating offspring BW to mother’s (offspring of female cohort members) and father’s (offspring of male cohort members) BW, showing the steeper and tighter intergenerational BW correlation through the matriline.

**Table 4:** Regression models predicting birth weights in offspring of female participants (n = 675).

	Model 1^a^	Model 2	p	Model 3	p	Model 4	p	Model 5	p
Mother’s birth weight (kg)		253 (53)	0.0001	286 (55)	0.0001	271 (58)	0.0001	−0.67 (1.46)	0.647
Mother’s gestational age at birth (weeks)				−24.9 (11.8)	0.035	−24.5 (11.8)	0.039	−24.2 (11.9)	0.042
Mother’s height (cm)						4.0 (4.9)	0.423	−14.3 (29.3)	0.625
Mother’s height–by-mother’s birth weight								0.006	0.520
Model adjusted R^2^	0.030	0.067		0.074		0.074		0.073	

Values are β (SE).

a Base model adjusts for mother’s age, first born, born early, born late, male baby, worked during pregnancy, prenatal care during pregnancy.

**Table 5:** Regression models predicting birth weight in offspring of spouses of male participants (n = 426).

	Model 1^a^	Model 2	p	Model 3	p	Model 4	p	Model 5	p
Father’s birth weight (kg)		127 (56)	0.024	145 (59)	0.014	132 (59)	0.017	1.13 (1.7)	0.493
Father’s gestational age at birth (weeks)				−14.2 (13.1)	0.280	−16.6 (13.4)	0.215	−17.3 (13.4)	0.198
Mother’s height (cm)						8.7 (5.2)	0.094	29.0 (32.4)	0.371
Mother’s height–by-father’s birth weight								−0.007 (0.01)	0.545
Model adjusted R^2^	0.033	0.043		0.044		0.051		0.049	

Values are β (SE).

a Base model adjusts for mother’s age, first born, born early, born late, male baby, worked during pregnancy, prenatal care during pregnancy.

While we can say with high certainty that the mothers in this study are the biological mothers of the children, paternity is less certain. False paternity would tend to cause an attenuation of father-offspring BW associations that could imitate a maternal effect [19]. To determine what rate of false-paternity would be necessary to explain the differences between mother-offspring and father-offspring BW associations noted here, we next ran a resampling analysis (see File S1 for details). Briefly, by systematically replacing the BW values of mothers with randomly selected mothers and then examining the new mother-offspring BW association, we simulated the effect of false paternity–or more precisely, what level of false-maternity would be required to achieve mother-offspring BW associations as low as the father offspring associations. By resampling 10,000 times each at systematically varying levels of false-maternity, we found that a false-maternity rate of 25% would be expected to attenuate the association enough to explain the differences between mother-offspring and father-offspring coefficients less than 2.5% of the time. A false maternity rate of 50% would be expected to explain the observed difference in associations 50% of the time (see File S1 for details). There is some evidence that women prefer men of relatively taller stature when pursuing short-term partners [25], [26], [27]. However, our simulations of such a non-random mating pattern show that this would tend to increase the father-offspring correlation rather than decrease it, suggesting that this is unlikely to explain the differences in parent-offspring BW correlations in fathers and mothers at Cebu (see File S1 for details).

**Figure 3:**
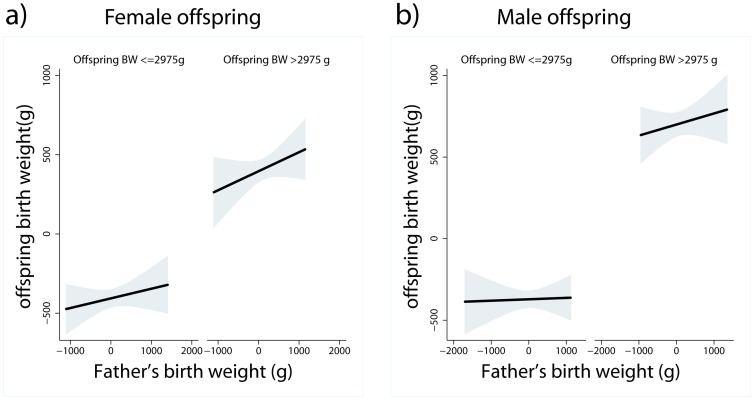
Best fitting regression lines (shaded area = 95% CI) relating offspring BW to the father’s BW, stratified on a median split of offspring BW in a) female offspring and b) male offspring. Father’s BW plotted as residuals adjusted for parent’s gestational age at birth. Offspring BW plotted as residuals adjusted for mother’s age, offspring’s gestational age at birth, first born status, offspring sex, prenatal care, and mother’s work status during pregnancy.

We next tested for evidence of maternal constraint in the sample using several strategies. First, following Veena et al [13] we investigated whether the father’s BW was a stronger predictor of offspring BW among heavier babies. Under the assumption that smaller newborns were more likely to have experienced growth restriction *in utero*, the hypothesis of maternal constraint leads to the expectation that genetic contributions to BW will be stronger in larger newborns. Because prior work on maternal constraint in humans and other non-human primates reported evidence for an effect specific to the matriline [20], [28], [29], we first stratified models on sex of offspring. All models included maternal adult height and the same confounding influences included in prior models. Contrary to expectations, the father’s BW × median split of offspring BW interaction was not significant in either female offspring (interaction p<0.539) or male offspring (interaction p<0.194, models not shown). Stratifying models on high/low offspring BW, paternal BW tended to be strongest as a predictor of offspring BW among heavier babies, with the relationship only approaching significance in the higher offspring BW stratum for male offspring (Table 6). Figure 3 shows the slopes linking offspring BW and paternal BW stratified on high/low offspring BW, and adjusting for the same potential confounding factors.

**Table 6:** Regression models relating offspring birth weight to father’s birth weight stratified on offspring sex and median split of offspring BW.

	FemaleOffspring(n = 210)	p-value	MaleOffspring(n = 220)	p-value
offspring BW ≤2975 kg	0.04 (0.05)	0.451	0.01 (0.05)	0.859
Model R^2^	0.180		0.079	
				
offspring BW >2975 g	0.10 (0.07)	0.135	0.11 (0.08)	0.143
Model R^2^	0.048		0.211	

Values are β (SE), models adjust for mother’s age, first born status, born early, born late, male baby, worked during pregnancy, prenatal care during pregnancy.

**Figure 4:**
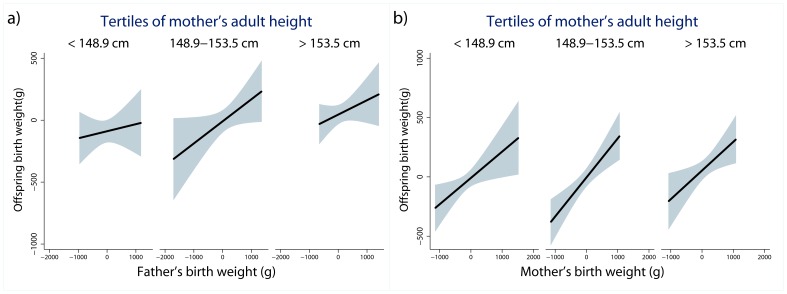
Best fitting regression lines (shaded area = 95% CI) relating a) offspring BW to father’s BW stratified on tertiles of mother’s adult standing height, and b) offspring BW to female participant’s BW stratified on tertiles of their own adult standing height. Parental BW plotted as residuals adjusted for parent’s gestational age at birth. Offspring BW plotted as residuals adjusted for mother’s age, offspring’s gestational age at birth, first born status, offspring sex, prenatal care, and mother’s work status during pregnancy. Adjusted regression coefficients (slopes) for each tertile of mother’s height (kg offspring BW/kg parental BW) = 0.06±0.10, 0.18±0.09*, 0.12±0.09 for father’s BW models, and 0.22±0.09*, 0.33±0.08*, 0.24±0.10* for mother’s BW models, *p<0.05.

Finally, we tested whether maternal and paternal BW were stronger predictors of offspring BW when the mother was taller (Table 4; Model 5), as would be expected if chronic early life nutritional stress or adult stunting places limits on genetic contributions to offspring fetal growth rate. Contrary to this expectation, the paternal and maternal height x parental BW interactions were both highly non-significant (both interactions p>0.6). These relationships are portrayed graphically in Fig. 4, which shows that the slopes of the linear trends relating offspring BW to and paternal and maternal BW were comparable across tertiles of the mother’s adult stature.

## Discussion

In this sample of Filipino young adults, we find evidence for stronger intergenerational BW correlations through the matriline than through the patriline, consistent with disproportionate maternal contributions to offspring BW. However, contrary to the concept of maternal constraint, there was no evidence that either patrilineal or matrilineal intergenerational BW correlations were stronger among taller women. Thus, there was no evidence that a woman’s history of nutritional stress, as reflected in her adult stature, constrain hereditary contributions to birth weight in this sample.

The finding of stronger maternal-offspring than paternal-offspring BW correlations has been described in most prior human studies. In a study of more than 67,000 Norwegian births, the correlation between mothers’ and offspring BW (r = 0.226) was stronger than that of the paternal BW-offspring BW correlation (r = 0.126) [12]. Similarly, Coutinho et al [14] found stronger effects of maternal BW on offspring BW compared to paternal BW among black (n>12,000) and white (n>100,000) residents of Illinois, while a greater maternal than paternal effect of SGA on SGA risk in offspring was reported in France [30]. In contrast to these studies, two studies in different regions of India have reported that paternal and maternal BW each predict comparable changes in offspring BW (n>500 ref. 13) with paternal BW even predicting a slightly (but not significantly) larger change in offspring BW (n>800 ref. [31]).

Although the pattern that we find is generally consistent with most past studies, little is currently known about the causes of the difference in strength of mother-offspring versus father-offspring BW correlations. Higher false paternity rates will tend to decrease the correlation between putative father and offspring for all traits with a genetic component [19], [32], and thus could contribute to, or account for, the greater associations between mother and offspring BWs than between father and offspring BWs. Our resampling testing suggested that false paternity rates of >25%, and most likely higher than 50%, would be required to explain differences in mother-offspring versus father-offspring BW associations of the magnitude that we find at Cebu (see File S1). While we are not aware of unbiased estimates of false paternity rates, in studies biased towards paternal confidence, false paternity rates average around ∼2–3%, while rates around 30% are found from studies in which confidence is low, such as from commercial paternity testing laboratories [33]. Based upon these figures, we feel that false paternity, at least on its own, is an unlikely explanation for the difference in relationships that we document between father-offspring and mother-offspring BW.

Other possible explanations for this pattern include sex-linked genetic effects, indirect genetic effects, epigenetic effects, and shared environmental or cultural effects. Although varying widely in mechanism, these explanations all share an intergenerational character. They all require that some environmental, physiological, or genetic factor which influences the mother *in utero* also tends to influence her child *in utero*. While these explanations are difficult to disentangle, here we consider some of the prime candidates in hopes of guiding future studies aimed at clarifying underlying mechanisms.

Fathers do not pass on their X chromosomes to sons but only to daughters, which results in a distinctive inheritance pattern for X chromosome-linked traits which in theory could in result discrepancies in patrilineal and matrilineal inheritance. However, consistent with previous large studies [12], [14], we found no difference in father-offspring BW associations depending on the sex of the child, which suggests that X chromosome-linked effects do not play an important role in explaining this pattern.

Another well-established sex-linked genetic effect which might explain excess matrilineal inheritance, mitochondrial inheritance, cannot be ruled out so readily. Mitochondria are present in the cytoplasm of cells, and contain their own 16,569 base-pair circular genome [34]. They are transmitted from mother to offspring via the egg, but are not transmitted from father to offspring. In support of a mitochondrial genetic contribution to BW, an analysis of a group of well-pedigreed captive Pigtailed Macaques (*Macaca nemestrina*) suggested that mitochondrial inheritance could account for 9% of the variance in BW [18]. Importantly, consistent with a durable mode of inheritance, this effect did not degrade with distance of relatedness. This is in contrast to expectations for non-genetic multi-generational maternal effects which are thought to have a more transient and environmentally-malleable nature [35].

Indirect genetic effects are another plausible contributor to excess matrilineal BW heritability. In contrast to mitochondrial effects or non-genetic maternal effects, indirect genetic effects occur because the presence of alleles in the offspring predicts the alleles of the mother and some of these alleles alter maternal physiology or metabolism, thereby influencing offspring phenotypes such as BW. As one concrete example, maternal alleles associated with type 2 diabetes influence the BW of offspring in part by influencing maternal fasting glucose and insulin levels during pregnancy [17]. Given the importance of the mother’s physiology and metabolism as an influence on fetal growth rate, and the lack of any paternal analogue to such effects, indirect maternal genetic effects are likely contributors to the finding of excess matrilineal BW heritability.

Environmental or cultural factors might also help explain the excess matrilineal BW heritability. Mothers and grandmothers often provide advice to their daughters and granddaughters about ideal behaviors during pregnancy [e.g 36,37,38], which could contribute to similarities in the gestational environments and birth weights of successive generations (shared family effects). Similarly, wealth is transmitted across generation, which could lead to similarities across generations in environmental factors like nutrition and access to health care [39], although it is not clear if this would contribute to larger mother-offspring correlations than father-offspring correlations. Experiences of discrimination on the basis of ethnicity or appearance might also lead parents and their offspring to experience similar levels of psychosocial stress across generations, which is known to contribute to poor birth outcomes [40], but is also not as obvious as an explanation for mother-father differences in the strength of intergenerational BW correlations.

Finally, there is also increasing evidence that the fetal growth conditions experienced by the mother, as reflected in her own gestational nutrient or hormonal milieu, can lead to durable epigenetic or developmental changes that influence offspring through several possible pathways. This can occur when the mother’s early life experiences influence her own fetal growth and also have durable effects on her adult physiology and metabolism that modify fetal growth rate of her offspring [40], [41]. At Cebu we recently reported that women with higher bedtime cortisol or who had a pro-inflammatory cytokine profile measured outside of pregnancy tend to give birth to smaller babies [42], [43]. Because lower birth weight has been shown to predict elevated adult cortisol and C-reactive protein in this and other samples [42], [44], these findings suggest that the mother’s own gestational environment could indirectly influence the gestational environment that she provides her offspring, thereby potentially helping explain the stronger mother-offspring birth weight heritability. Because such “soma-to-soma” effects require direct interaction between parental and offspring phenotype, they may only be transmitted via the matriline. In addition, there are also a growing list of examples of environmentally-induced epigenetic changes that are transmitted directly via sperm or egg to offspring and even grandoffspring, which are not limited to matrilineal inheritance [45].

Regardless of the specific causes of the differences in matrilineal and patrilineal BW inheritance observed here, our findings, and those of previous investigators, clearly show that maternal contributions are more important than paternal contributions in determining offspring BW. Because prenatal nutrition and growth rate predict an expanding array of long-term functional and health outcomes [40], [46], [47], it will be important for future research to disentangle the mechanistic basis for this excess in matrilineal BW heritability. Based upon our findings and the above review, indirect maternal genetic effects, maternal mitochondrial effects, and long-term epigenetic or developmental effects of the mother’s early life experience appear to be among the most promising candidate influences.

Some limitations of this study warrant consideration. Most notably, offspring BW information was recalled by women during interviews, rather than being prospectively collected after each pregnancy. In Cebu, most women have birth records, and analyses were limited to births that were weighed. Although this will minimize recall error, female cohort members were asked about their reproductive histories during several survey rounds, while the spouses of male participants were asked only once in 2009. Thus, it is possible that female participants were better able to recall, or have records of, their offspring’s BW, which could reduce measurement error among the offspring of female participants. However, we found that maternal socioeconomic and pregnancy characteristics were as strong or often stronger as predictors of offspring BW among spouses of male participants, suggesting that the apparently weaker relationship between paternal BW and offspring BW was not simply an artifact of poor measurement reliability of BW among offspring of male participants.

### Conclusion

In conclusion, mother’s BW is a stronger predictor of offspring BW than is the father’s BW in this population, independent of the mother’s adult size. This suggests that some combination of maternal indirect genetic, environmental or epigenetic factors influence the intrauterine environment or fetal growth above and beyond any direct genetic effects. Although this contributes to an excess of BW heritability through the matriline, we found no evidence that the slope of intergenerational BW relationships were steeper or stronger in heavier offspring or among newborns born to taller women, as would be predicted if maternal nutritional stress or stunting constrained genetic contributions to birth size. These findings suggest that maternal effects on fetal growth are present across the full range of maternal stature in this population. By extension, they suggest that maternal environmental, epigenetic or indirect genetic factors influence the diverse array of offspring phenotypes that are downstream of fetal nutrition and growth.

## Supporting Information

File S1 **Non-paternity analysis.**
(DOC)
