# Supplementary material for: Intergenerational Predictors of Birth Weight in the Philippines: Correlations with Mother’s and Father’s Birth Weight and Test of Maternal Constraint
Source: PLoS One. 2012 Jul 27;7(7):e40905. doi: 10.1371/journal.pone.0040905 (PMC3407139; doi:10.1371/journal.pone.0040905)
Supplement: File S1 — Non-paternity analysis. (DOC) [file pone.0040905.s001.doc]

**Supplementary File 1:**

False paternity might be a cause of the greater β coefficient, or stronger correlations, relating parental birth weight (BW) with offspring (BW) in mothers compared to fathers. This is because measurement error of independent variables is known to cause attenuation of beta coefficients in ordinary-least-squares regression[^1 pg 325^](#_ENREF_1). Falsely assuming a socially recognized father is a biological father creates such a measurement error.

To test this possibility as an explanation for the evidence for greater matrilineal heritability in BW, we used a randomization technique to test for the expected effects of various levels of paternal uncertainty on β coefficient estimates. Analyses were conducted in R (version 2.14.0; see below for code). We started with the maternal-offspring BW regression from Table 5, Model 2 in the main manuscript (‘Births to female participants’). Since we have a high certainty that the socially recognized mother is the biological mother, we can randomly re-assign a fraction of the mothers with BWs from other mothers to simulate the effects of false paternity. Of the 675 mother-offspring pairs, we started by randomly selecting 5% of the maternal BW values and replacing these maternal BW values with maternal BW values from other randomly selected mothers (without replacement). Then, the regression from Table 5, Model 2 was run and the β coefficient for Mother’s BW was stored. This process was repeated 10,000 times and from these 10,000 β coefficients, the 2.5^th^, 50^th^, and 97.5^th^ centiles were calculated. This process was then repeated for increasing percentages of the 675 mother-offspring pairs.

Supplementary Figure 1, below, summarizes the results. The blue dotted line represents the father-offspring β coefficient observed in the male participants (Table 5, Model 2), while the y-intercept of the black line represents the β coefficient for the female participants in the same model. At the y-intercept of 0% randomization, or 0% false parents, the β coefficients are identical to Table 5, Model 2. As the fraction of the sample that is randomized is increased, simulating greater false paternity rates, the β coefficients decrease. These results suggest that the best estimate of the frequency of false paternity that would completely explain the differences in β coefficients between mother-offspring and father-offspring BWs is ~50%. At a 25% false paternity, we would expect to see the father-offspring BW β coefficient reduced enough to explain the difference that we document only ~2.5% of the time.


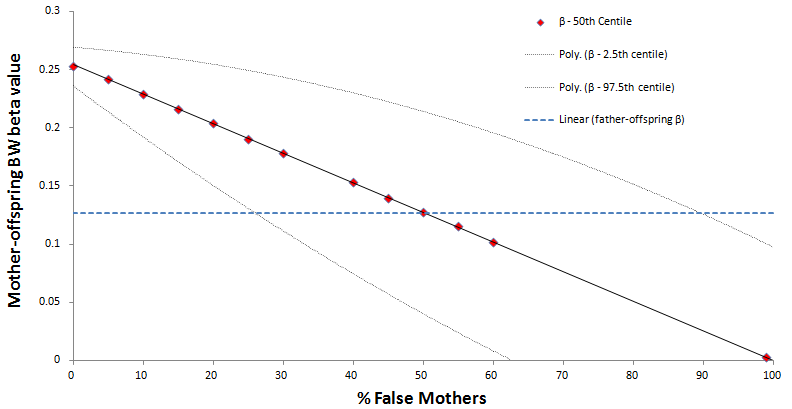


**Supplementary Figure 1**. Simulated effect of false paternity on parent-offspring birth weight β coefficients. Blue dotted line represents father-offspring β coefficient. Each red dot represents the 50^th^ centile (median) β coefficient of 10,000 permutations at a given % “False Maternity” rate with a black linear best fit line marking the 50^th^ centile and gray 2^nd^ order polynomial best fit lines for the 2.5^th^ and 97.5^th^ centiles. The intersection of black and blue lines at ~50% suggests that the best estimate of the degree of false paternity necessary to cause the observed difference between mother-offspring and father-offspring β coefficients is 50%.

This randomization model assumes that there are no systematic phenotypic or genetic differences between biological fathers and social fathers. However, we know from other studies that shorter males exhibit more jealousy and decreased relationship satisfaction.[^2^](#_ENREF_2) Correspondingly, women tend to find partner physical attractiveness (including increased height) more important when imagining selecting extra-pair short term relationships than long term relationships. Based on this, we expect true biological fathers will tend to be taller than socially recognized fathers. Since height is correlated with BW (r = 0.21, p < 0.0001), this effect of biological fathers being systematically taller could cause a bias in father-offspring associations. To test whether this bias could account for the differences in mother-offspring and father-offspring β coefficients we used another permutation procedure. We used the same strategy as the permutation testing described above, but with a modification that emulated the biological father being taller than the socially recognized father (see below for R code). For a subset of the randomized mothers, their BW values were only replaced with randomly selected BWs from mothers who were the same height or shorter than her. This process was repeated 1,000 times. As illustrated in Supplementary Figure 2, this procedure resulted in *higher* β coefficients than simple randomization in all tested cases. This suggests that women choosing biological fathers who are taller than the socially recognized father is unlikely to explain the differences in β coefficients observed at Cebu.

*
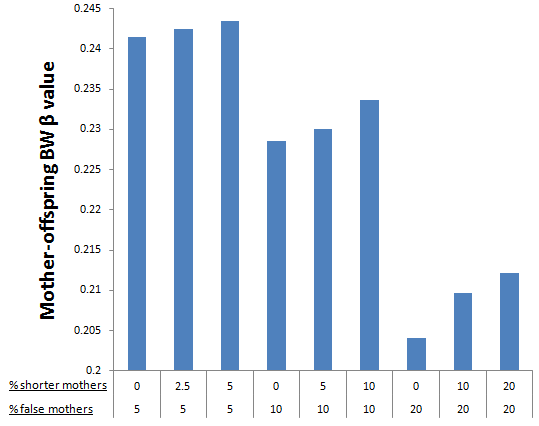
*

**Supplementary Figure 2**. Simulated effect of biased false paternity in situation in which biological father is taller than socially recognized father.

**Literature**

1. Greene WH. *Econometric analysis*. 6th ed. Upper Saddle River, N.J.: Prentice Hall; 2008.

2. Brewer G, Riley C. Height, Relationship Satisfaction, Jealousy, and Mate Retention. *Evolutionary Psychology* 2009;7(3): 477-89.

3. Scheib JE. Context-specific mate choice criteria: Women's trade-offs in the contexts of long-term and extra-pair mateships. *Personal Relationships* 2001;8(4): 371-89.

4. Pawlowski B, Jasienska G. Women's preferences for sexual dimorphism in height depend on menstrual cycle phase and expected duration of relationship. *Biological Psychology* 2005 Sep;70(1): 38-43.

**R code for randomization procedures:**

#Procedure 1

#Set to randomize 45 percent of cases or 675 * 0.45, which rounds up to 304 cases. Replacing 304 with other numbers allows randomization of varying percentages of randomized cases

#set-up place to store results with 10,000 rows for 10,000 tries

results <- matrix(nrow=10000,ncol=length(model$coef))

#looping 10,000 times

for (i in 1:10000) {

#bw is dataset

simdata <- bw

bwpermutation <- simdata[sample(1:nrow(simdata), nrow(simdata),

replace=FALSE),]

selectedcases <- bwpermutation[1:304,]

selectedcases$bestbw <- sample(selectedcases$bestbw,

nrow(selectedcases), replace=FALSE)

bwpermutation$bestbw[1:304] <- selectedcases$bestbw

results[i,] <- lm(iccbw ~ momyr + bornearly + bornlate + primipicc + male + pregwork + prenatal + bestbw, data=bwpermutation)$coef

if (i %% 1000 == 0) print(i)

}

#to get 50^th^, 0.025^th^ and 0.975^th^ centiles

summary (results)

quantile(results[,9], 0.025)

quantile(results[,9], 0.975)

#Procedure 2

# Changes 34 cases total (5% of 675) – but make so a fraction of these cases are re-assigned to shorter individuas.

# 1-17 get assigned bestbw from shorter person

# bestbw values for 18-34 randomly reordered

#1000 permutations

results <- matrix(nrow=1000,ncol=length(model$coef))

for (i in 1:1000) {

simdata <- bw

bwpermutation <- simdata[sample(1:nrow(simdata), nrow(simdata),

replace=FALSE),]

#1-17 get someone equal or shorter

selectedfirsthalf <- bwpermutation[1:17,]

#18-34 get someone else random

selectedsecondhalf <- bwpermutation[18:34,]

selectedsecondhalf$bestbw <- sample(selectedsecondhalf$bestbw,

nrow(selectedsecondhalf), replace=FALSE)

for (s in 1:17) {

cupcake <- bw

caseheight <- selectedfirsthalf$height[s]

cupcake$height <- ifelse(caseheight < cupcake$height, NA, cupcake$height)

cupcake <- na.omit(cupcake)

selectedfirsthalf$bestbw[s] <- sample(cupcake$bestbw, 1)

bwpermutation$bestbw[1:17] <- selectedfirsthalf$bestbw

bwpermutation$bestbw[18:34] <- selectedsecondhalf$bestbw

results[i,] <- lm(iccbw ~ momyr + bornearly + bornlate + primipicc + male + pregwork + prenatal + bestbw, data=bwpermutation)$coef

}

}

summary (results)

quantile(results[,9], 0.025)

quantile(results[,9], 0.975)
